# Cough flows as a criterion for decannulation of autonomously breathing patients with tracheostomy tubes

**DOI:** 10.1186/s12931-024-02762-w

**Published:** 2024-03-18

**Authors:** Jingyi Ge, Guangyu Niu, Qing Li, Yi Li, Bo Yang, Haiming Guo, Jianjun Wang, Bin Zhang, Chenxi Zhang, Ting Zhou, Zhanqi Zhao, Hongying Jiang

**Affiliations:** 1 Department of Respiratory Rehabilitation Center, Beijing Rehabilitation Hospital of Capital Medical University, Beijing, China; 2 School of Biomedical Engineering, Guangzhou Medical University, Guangzhou, China; 3 Institute of Technical Medicine, Furtwangen University, Villingen-Schwenningen, Germany

**Keywords:** Decannulation, Cough peak flow, Peak expiratory flow, Prolonged tracheostomy

## Abstract

**Background:**

Adequate cough or exsufflation flow can indicate an option for safe tracheostomy decannulation to noninvasive management. Cough peak flow via the upper airways with the tube capped is an outcome predictor for decannulation readiness in patients with neuromuscular impairment. However, this threshold value is typically measured with tracheotomy tube removed, which is not acceptable culturally in China. The aim of this study was to assess the feasibility and safety of using cough flow measured with tracheostomy tube and speaking valve (CF_SV_) > 100 L/min as a cutoff value for decannulation.

**Study design:**

Prospective observational study conducted between January 2019 and September 2022 in a tertiary rehabilitation hospital.

**Methods:**

Patients with prolonged tracheostomy tube placement were referred for screening. Each patient was assessed using a standardized tracheostomy decannulation protocol, in which CF_SV_ greater than 100 L/min indicated that the patients’ cough ability was sufficient for decannulation. Patients whose CF_SV_ matched the threshold value and other protocol criteria were decannulated, and the reintubation and mortality rates were followed-up for 6 months.

**Results:**

A total of 218 patients were screened and 193 patients were included. A total of 105 patients underwent decannulation, 103 patients were decannulated successfully, and 2 patients decannulated failure, required reinsertion of the tracheostomy tube within 48 h (failure rate 1.9%). Three patients required reinsertion or translaryngeal intubation within 6 months.

**Conclusions:**

CF_SV_ greater than 100 L/min could be a reliable threshold value for successful decannulation in patients with various primary diseases with a tracheostomy tube.

**Trial registration:**

This observational study was not registered online.

## Introduction

Tracheostomy remains one of the most commonly performed surgical procedures in the intensive care unit [1], and tracheostomy decannulation is an important step in the rehabilitation of patients recovering from a critical illness [2]. Assessment of readiness for tracheostomy decannulation could improve the rate of successful decannulation [3], and having an insufficient cough strength plays a major role in decannulation failure [4, 5]. Cough strength was assessed clinically by measuring expiratory muscle strength, cough peak flow (CPF) and cough reflex [6]. Bach and Saporito found that the ability to generate a CPF of at least 160 L/min after decannulation is necessary for the procedure to be successful in patients with neuromuscular disease irrespective of their ability to breathe [7]. They evaluated 22 patients with spinal cord injury (SCI) and 16 patients with global alveolar hypoventilation. However, the CPF of many patients without a tracheotomy tube, especially SCI patients, was less than 160 L/min [8]. Besides, various types of cough flows exist, including unassisted spontaneous voluntary or reflex cough flows, flows assisted by techniques like air stacking and abdominal thrusts, and flows triggered by mechanical insufflation-exsufflation (MI-E). The effectiveness of a cough is directly proportional to the magnitude of the cough or exsufflation flow. Besides, this threshold value is typically measured with tracheotomy tube removed. Due to the cognitive and cultural differences regarding tracheotomy and decannulation, most of the patients and their families in China cannot accept decannulation evaluation with the tracheotomy tubes removed: they cannot accept the scenario when the patients do not pass the evaluation, an underestimation they return the tracheotomy cannula again. Leak around the outer walls of the tracheotomy tube may lead to an underestimation of CPF, which may cause patients who are candidates for decannulation to continue tracheotomy cannulation for an unnecessarily long time. In another study [9], 23 decannulation attempts were made, and the majority of the subjects had a CPF less than 160 L/min, with an average of 99 L/min. No decannulation failed at 72 h. However, patients with neuromuscular disorders were excluded in that study.

In our department, patients with prolonged placement of a tracheostomy tube were referred from other general hospitals. Patients who are unable to wean from ventilators due to profound respiratory muscle weakness and poor cough flows may be decannulated to non-invasive ventilation, provided that MI-E exsufflation flows exceed 150 to 200 L/m, irrespective of their unassisted cough flow rate. In cases of non-ventilated patients, a multidisciplinary pulmonary rehabilitation team evaluates their condition using an established tracheostomy decannulation protocol that has been previously published [10]. With respect to the patient’s consciousness and cognitive level, cough flow measured with tracheostomy tube and speaking valve (CF_SV_) was performed. We define CF_SV_ greater than 100 L/min as a good indicator that the patient has adequate cough or exsufflation flow to decannulate. If the criterion is not met, the patient will first undergo cough augmentation techniques until the criterion is met and then decannulate; otherwise, the patient will remain with a tracheotomy tube for a further period. We also taught patients and caregivers how to assist cough at home after decannulation.

The aim of this study was to assess the feasibility and safety of CF_SV_ >100 L/min as a criterion for successful decannulation and to standardize the measurement methods so that they can be used routinely in the decannulation processes.

## Methods

### Patients

This prospective cohort study was approved by the ethics committee of Beijing Rehabilitation Hospital of Capita Medical University (2018bkky-121). The study was conducted in accordance with the Declaration of Helsinki. Informed consent was given by the next of kin, if available, and from the patients upon recovery of competency, in compliance with Chinese law. All patients with prolonged placement of a tracheostomy tube from January 2019 to September 2022 were screened for the study. Patients ready for the institutional decannulation protocol were included. The following variables recorded at inclusion in the study were demographics, Acute Physiology and Chronic Health Evaluation II (APACHE II) scores, Glasgow Coma Scale (GCS), primary disease, tracheostomy indication, tracheostomy time before referral, mechanical ventilation (MV) time before referral, and hospital length of stay before referral. The primary disease was categorized as pulmonary disease, multiorgan failure, acute brain injury (ABI), ventilatory pump failure (VPF), or thoracoabdominal surgery.

### Decannulation protocol

The institutional standardized tracheostomy decannulation protocol is as follows (see reference [10] for the detailed process):

#### Step 1

Confirming the patient’s clinical stability including being (1) weaned from a ventilator more than 48 h prior; (2) no organ failure; (3) no sepsis; (4) having a stable heart rate and blood pressure without the use of vascular active drugs; (5) any lung infection under control; and (6) a PaCO_2_ < 60 mmHg.

#### Step 2

Tolerance to the speaking valve for at least half an hour (Covidien, Italy). The aim of this step is to evaluate the patency of the upper airway. CF_SV_ is measured. According to the patient’s consciousness and cognitive level, the modalities of assessment were as follows: clinical assessment and CF_SV_, including PCF (Keka, Shanghai) and PEF (Jaeger, MasterScreen, Germany). If the CF_SV_ value was lower than 100 L/min, pulmonary rehabilitation was performed (see the “Cough augmentation techniques” section for further details).

#### Step 3

Continue wearing the speaking valve for 4 h, and no tracheostomy cannula is used for sputum suction within these 4 h.

#### Step 4

Readiness for decannulation. Evaluating CF_SV_ again before decannulation. If the CF_SV_ value was higher than 100 L/min, the patient was decannulated.

## Measurement of CF_SV_

The following describes the details of the CF_SV_ measurement procedures.

### Preparation

Because there are many patients with dysphagia in our department, tracheotomy tubes with cuffs are used (Smiths Medical), and aspiration under the glottis is often performed. After oral and nasal secretions have been suctioned, the cuff is deflated. Then, the speaking valve was placed so that supplemental oxygen could be provided from the side hole of the speaking valve if needed.

### Position

The patient is in a sitting position. If the patient could not complete the measurement, he or she was positioned supine with the head of the bed elevated at 30°.

### Measurement

*Voluntary cough*: If the patient can cough on command, CF_SV_ is measured from a voluntary cough through the mouth with a mechanical peak flow meter when wearing a speaking valve (Keka, Shanghai).

*Induced cough*: If the patient cannot cough on command due to lack of consciousness or poor cognitive state, the CF_SV_ is measured from an induced cough. The bed is tilted for at least 45 degrees and patient’s head and neck are kept in a neutral position with the chin slightly elevated. Airway secretion is cleared. The suction catheter is inserted through the leak port and the flow meter is connected to the patient’s tracheostomy tube using a T-tube. While the nurse stimulates the airway, and the therapist records the test values. The average values of 2–3 tests is used.

All measurement procedures in the present study were conducted by one physiotherapist. The vital signs were monitored in room air without O_2_ supplement.

### Number of measurements and time points

When the patient fully understood the procedure, the best value of at least three consecutive measurements was recorded, with a 2-min rest in between.

The best value after 30 min of wearing the speaking valve, the best value before decannulation and the best value 5 days after decannulation were recorded.

### Precautions

Oxygen saturation and vital signs were monitored during the measurement process.

## Cough augmentation techniques

If the CF_SV_ value was lower than 100 L/min, cough augmentation rehabilitation techniques were individualized by the physiotherapists after assessment. Cough augmentation techniques consist of lung volume recruitment (also termed air stacking or breath stacking and rib bounce technique) [11]; inspiratory muscle strength training (Power Breath KH2, load set in 55-65% MIP, ten times per group, 3 groups, twice per day), manually assisted cough (abdominal compression at the end of exhalation, thoraco-abdominal combined with auxiliary airway clearance) [12], mechanically assisted cough using a MI-E device (Cough Assist TM Philips, E70) [13], respiratory muscle strength treatment (including abdominal muscle resistance strength training, core strength training and trunk rotation training) and exercise training (rehabilitation treadmill training, upper limb ergometer training, in situ step training and walking training with supplemental oxygen or not).

## Endpoints

The primary outcome was the success rate of decannulation of patients who met the objective assessment criteria for cough strength. Decannulation failure was defined as requiring reinsertion of the tracheostomy tube within 48 h after decannulation. Secondary outcomes of interest were the reintubation and mortality rates after 6 months of follow-up. Exploratory outcomes included (1) the characteristics of patients with CF_SV_ over 160 L/min and between 100 and 160 L/min in the successful decannulation group; (2) changes in CF_SV_ values at different time points in patients with different primary diseases; and (3) CF_SV_ improvements after pulmonary rehabilitation in patients who had initially low CF_SV_.

### Statistical analysis

Data are presented as the mean ± standard deviation (SD) or median [interquartile range (IQR)] for continuous variables and as the frequency (%) for categorical variables, where appropriate. Student’s t test or the Wilcoxon rank sum test was used for continuous variables, and the χ2 test or Fisher’s exact test was used for categorical variables. The bar chart was used to show the distribution of CF_SV_ of different diseases at three time points.

All statistics were two sided, with *P* < 0.05 considered significant. Statistical analysis was performed using SAS version 9.4 (SAS Institute Inc., Cary, NC).

## Results

### Participants

**Fig. 1:**
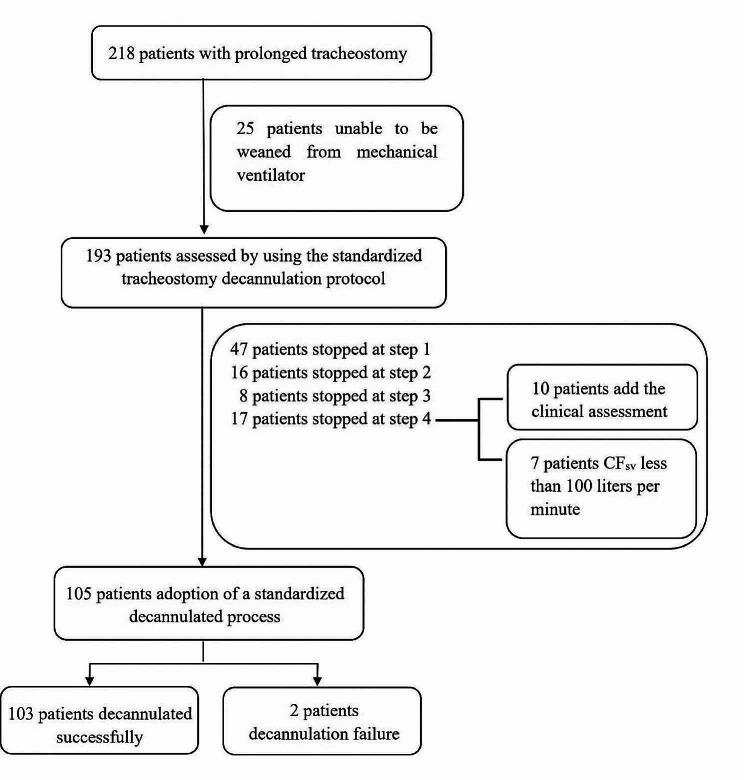
Flowchart of patient inclusion

Two hundred eighteen patients with prolonged placement of tracheostomy tubes were screened. Twenty-five patients were unable to be weaned from mechanical ventilation successfully. In the end, 193 patients were included in the study and assessed by using the standardized tracheostomy decannulation protocol (Fig. 1). The size of the tracheostomy tubes depended on the height and weight of the patients (No. 8 in 19 patients, No. 7.5 in 118 and No. 7 in 56; all from Smiths Medical with deflated cuff). Eight patients died within 2 weeks. The causes of death were: acute septic cholangitis leading to sepsis in one case, infection with novel coronavirus in two cases, progression of primary disease (e.g., tumor, autoimmune disease, etc.) in three cases, and recurrent cerebral hemorrhage in two cases. A total of 80/185 patients did not meet the criteria for decannulation. Among them, 39 patients (48.75%) were unable to progress further than step 1 (unstable clinical status), 16 patients (20%) stopped at step 2 (could not tolerate wearing speaking valves for 30 min), 8 patients (10%) stopped at step 3 (unable to tolerate speaking valve for 4 h), and 17 patients (21.25%) stopped at step 4 (2nd cough ability assessment) by the end of the study. One hundred five patients were decannulated successfully, and 2 patients underwent reinsertion (failure rate 1.9%). The demographic and clinical characteristics of the patients who were successfully decannulated and those who failed the protocol are shown in Table 1. Sex, age, primary disease, tracheostomy time, MV time and length of hospital stay did not differ between two groups.

**Table 1:** Demographics of patients included in the study

Characteristic	Met the decannulation protocol criteria (*n* = 105)	Did not meet the decannulation protocol criteria (*n* = 88)	P value
Age, years			0.921
Mean ± SD	63.38 ± 15.94	64.67 ± 11.45	
Sex (%)			0.443
Male	135 (70.48)	70 (80.00)	
Female	58 (29.52)	18 (20.00)	
Primary disease (%)			0.257
Pulmonary disease	44 (22.86)	6 (6.67)	
Acute brain injury	83 (42.86)	53 (60.00)	
VPF	35 (18.13)	29 (32.95)	
Thoracoabdominal surgery	27 (13.99)	0 (0.00)	
Multiorgan failure	4 (2.07)	0 (0.00)	
Tracheostomy time before referral – days (IQR)	68.4 (72.6)	76.6 (78.8)	0.427
Mechanical ventilation time before referral -days (IQR)	30.2 (42.4)	38.3 (50.7)	0.265
Length of hospital stay before referral -days (IQR)	79.0 (73.5)	84.1 (80.3)	0.129

VPF, ventilatory pump failure; IQR, interquartile range

The details of two reinsertion cases are as follows: One case was a 46-year-old patient with SCI. MI-E was utilized to effectively clear his secretions and a drop in oxygen saturation was promptly addressed by maintaining it above 95% after decannulation. The oxygenation index, as indicated by repeated blood gas analysis, exceeded 300 mmHg. However, his psychological fear and insecurity greatly contributed to the decision of re-inserting the tracheotomy tube. Another case of reinsertion was an 85-year-old patient, the decision to re-insert the tube was prompted by the presence of tracheal tenderness and airway collapse at the site of the tracheotomy cannula, which unfortunately could not be detected during our decannulation process. This outcome was a result of structural damage to the airway caused by cartilaginous ring fracture resulting from the tracheotomy. In such cases, noninvasive positive pressure ventilation alone would not effectively deliver air into the lungs.

### Follow-up of 6 months

Within the 6-month follow-up period, 1 patient in the decannulation group was reintubated due to COVID-19. Two patients died. An 80-year-old male patient died due to acute gallbladder necrosis and peritonitis 1 month after decannulation. The other patient was intubated 2 months after decannulation due to the aggravation of pulmonary infection. Subsequently, his family gave up after exacerbation, and he died. The remaining 100 patients did not undergo reintubation, the incidence of pneumonia was 4.8% (*n* = 5), and the readmission rate was 6.7% (*n* = 7).

Of the patients who did not meet the criteria for decannulation, 3 patients died within 6 months of follow-up (*P* = 0.52).

### Comparison of patients in the decannulation group with CF_SV_ values between 100 and 160 L/min to patients with values > 160 L/min

The patients in the successful decannulation group were further divided into CF_SV_ between 100 and 160 L/min and > 160 L/min (Table 2). There were no significant differences in the baseline characteristics or the outcomes within the follow-up period.

**Table 2:** Baseline characteristics of the successfully decannulated population under different CF_SV_ subgroups before decannulation

Characteristic	Before decannulation CF_SV_ 100-160 L/min (*n* = 94)	Before decannulation CF_SV_ >160 L/min (*n* = 11)	P value
Age, years			0.92
Mean ± sd	63.33 ± 16.46	63.82 ± 11.06	
Sex (%)			0.17
Male	64 (68.09)	10 (90.91)	
Female	30 (31.91)	1 (9.09)	
Primary disease (%)			0.38
Pulmonary disease	22 (23.4)	2 (18.18)	
Acute brain injury	41 (43.62)	4 (36.36)	
VPF	18 (19.15)	1 (9.09)	
Thoracoabdominal surgery	11 (11.7)	4 (36.36)	
Multiorgan failure	2 (2.13)	0 (0.00)	
Reintubation (%)			>0.99
No	93 (98.94)	11 (100)	
Yes	2 (1.06)	0 (0)	
Death (%)			0.20
No	93 (98.94)	10 (90.91)	
Yes	1 (1.06)	1 (9.09)	

### Comparison of CF_SV_ values in patients with different primary diseases

**Fig. 2:**
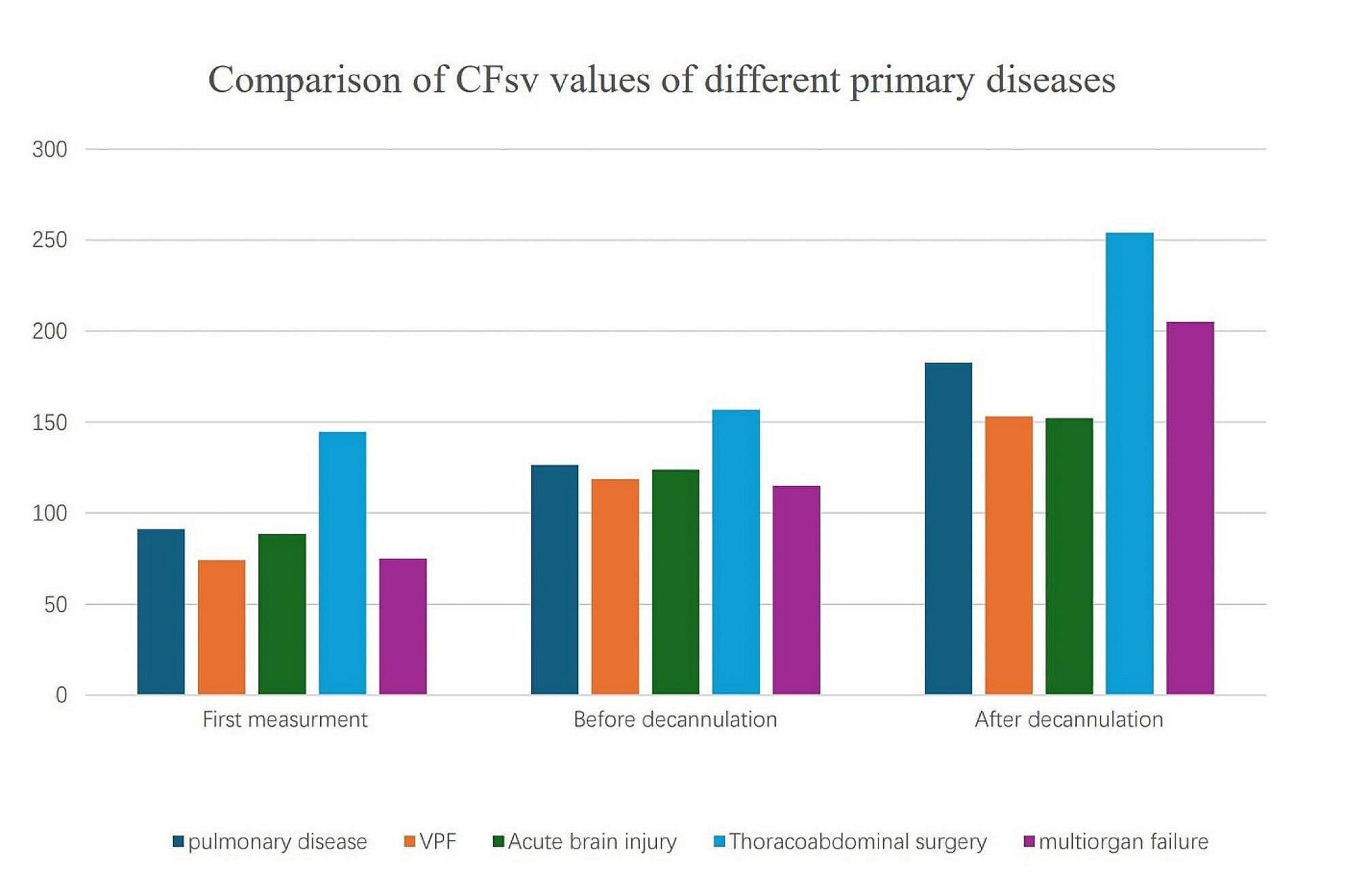
CF_SV_ values in patients with different primary diseases

The mean CF_SV_ values of the first measurement, before decannulation and after decannulation in patients with different primary diseases are shown in Fig. 2. The highest values were found in the thoracoabdominal surgery group, with mean values of 144.67 ± 62.26 L/min, 156.67 ± 54.34 L/min and 254 ± 81.02 L/min. The lowest values were in the VPF group, with mean values of 70 ± 36.06 L/min, 116.67 ± 5.77 L/min and 153.33 ± 20.82 L/min (*P*<0.005), however with MIE these flows might have been much greater.

To assess the air leakage, the peak flow differences between post-decannulation and our setup with tracheostomy tubes in and speaking valve were compared for all patients who were successfully extubated (Table 3). In patients with multiorgan failure, and thoracic and abdominal surgery, the air leak was relatively large, whereas for patients with pulmonary diseases, acute brain injury and VPF, the leak was small.

**Table 3:** Cough flow with and without tracheostomy tube

Primary disease *N* = 103	before decannulation (L/min)	After decannulation (L/min)	Differences (L/min)
Pulmonary disease (*n* = 24)	126.7 ± 22.9	176.5 ± 74.4	49.8 ± 61.0
Acute brain injury (*n* = 45)	124.7 ± 25.5	152.7 ± 38.1	28.0 ± 28.1
VPF(*n* = 18)	119.2 ± 23.7	154.2 ± 32.3	35.0 ± 22.6
Multiorgan failure (*n* = 1)	115	205	90
Thoracoabdominal surgery (*n* = 15)	161.3 ± 53.7	243.7 ± 81.3	82.3 ± 63.5

### Comparison of CF_SV_ values before and after pulmonary rehabilitation

Eighty patients failed to reach the standard in the first cough ability assessment (average value was 73.54 L/min), including 23 patients with VPF, 38 with ABI, 17 with pulmonary disease and 2 with thoracoabdominal surgery. Physiotherapists conducted cough enhancement training with the patients based on their individualized assessment. Among them, 34 patients underwent chest expansion and artificial inflation due to the decrease in lung volume caused by the primary disease, and 26 patients were given abdominal muscle group training due to the decrease in expiratory muscle strength. For twenty SCI patients, MI-E was added to simulate normal cough and increase cough ability. After an average of 26.0 ± 3.2 days of pulmonary rehabilitation, the average CF_SV_ values of 73 patients increased to the threshold value, with a mean value of 119.44 ± 14.38 L/min. These patients were then decannulated successfully. However, 7 patients still did not reach the standard and were not recommended for decannulation but for tracheotomy cannula maintenance, including 6 with VPF, and 1 with ABI.

## Discussion

This prospective study suggested that as a means of measuring cough capacity, a CF_SV_ value greater than 100 L/min could be a reliable criterion for successful decannulation in patients with prolonged placement of a tracheotomy tube due to various primary diseases. Only 2 patients required reintubation after completing the institutional decannulation protocol. Another 3 patients were intubated within the 6-month follow-up period. To the best of our knowledge, this is the first study using CF_SV_ prospectively as a part of the protocol to guide the procedure of decannulation rather than perform a retrospective analysis of the relationship between cough ability and the success of decannulation [14]. Nevertheless, the threshold 100 L/m was proposed and evaluated with the tracheostomy tubes and speaking valve. However, the actual flows may have approached 150 L/m or more due to leak around the tube.

Assessment of readiness for decannulation is an essential step of the tracheostomy decannulation process [15]. An international survey showed that clinicians rated patient level of consciousness, ability to tolerate tracheostomy tube capping, cough effectiveness, and secretions as the most important factors [3]. These factors are extremely important when non-invasive ventilation support or MI-E is not in use. There is broad consensus that voluntary cough efficiency is one of the most effective criteria for decannulation [16]. A weak cough was previously identified as a risk factor for reintubation [17]. Most studies used subjective cough assessment methods, including effective cough and voluntary or evoked cough for cognitively impaired subjects. Singh et al. suggested that objective assessment could be superior in various patients [16]. The CPF value in healthy subjects exceeded 360 to 400 L/min, whereas that for mucus expectoration should exceed 160 to 200 L/min [18, 19]. In patients with neuromuscular disease, CPF > 250–270 L/min could be sufficient to prevent pneumonia [6, 20]. After comparing several independent variables, Santus et al [21]. proposed a quantitative semiquantitative score, the QsQ score, including the objective quantitative parameters of cough effectiveness and the ability to tolerate tracheostomy tube capping. However, this score has never been validated in actual clinical applications.

Bach and Saporito found that only the ability to generate a CPF of at least 160 L/min predicted the success of decannulation [7]. The authors hypothesized that the ability to create expiratory airflow to clear secretions may be an important parameter for determining when it might be safe to extubate or decannulate patients and whether they require ventilatory assistance. For patients who had undergone thoracoabdominal surgery or had severe pneumonia, heart failure and other diseases, their cough ability was not affected by the primary diseases, so it was easy to reach the standard of 160 L/min. The Bach and Saporito study was conducted before MI-E devices had the ability to measure effective cough flow. Nowadays, CPF assisted or unassisted can be used to determine need for tracheotomy or removal. CPF was usually measured with removing the tracheostomy tube to prevent possible leak [22]. Such leak would lead to an underestimation of the cough flow. However, in China, due to the educational and cultural differences, most of the patients and their families cannot accept decannulation evaluation with the trach tubes removed. The leak varies among patients depending on the diseases (Table 3). We suspect that there are other countries and cultures similar to China, which cannot accept the evaluation without trach tubes (not practically but emotionally). Therefore, it is essential to establish an CF_SV_ threshold value for clinical centers facing the same issues. In our study, a CF_SV_ greater than 100 L/min was used according to our clinical experience. The results of our study confirm that this CF_SV_ measurement with the proposed threshold is reliable compared with other parameters.

The patients referred to our department had high-level SCI, scoliosis after orthopedic surgery, ABI, and decreased CF_SV_ values due to denervation of abdominal muscles, decreasing lung volume, etc. SCI patients most commonly have decreased cough ability (when measuring with tracheostomy tube). The average first measurement was 75 ± 10.33 L/min, and in the decannulation group, the value before decannulation was 119.06 ± 24.91 L/min and that in the group not recommended for decannulation was 70 ± 18.32 L/min, which was lower than that in other studies [23, 24]. Whether the CF_SV_ was measured before or after decannulation makes a difference. Because the tracheostomy tube itself in the airway increased the respiratory work and the ostomy could be covered to eliminate leak, CF_SV_ increased after decannulation. Therefore, the threshold set before decannulation should be lower by at least 34.5 L/min [23]. Therefore, a CPF of 130 L/min before decannulation was used as a threshold in a previous study [23]. In our center, CF_SV_ is measured from a voluntary cough or induced cough, without manual assistance, which is one of the reasons why the threshold in our decannulation protocol is much lower than that proposed in other studies. It was suggested that the assisted cough flow should be assessed instead, since the patients could be supported by assisted ventilation or use MI-E for airway clearance even after decannulation [25]. The objective of our study was to establish a threshold with tracheostomy tube for decannulation. Since the training/experience levels of the physiotherapists could be very different from site to site, the assisted cough flow might vary so that it would be hard to generalize such threshold. Nevertheless, for clinical practice, assisted coughing is applied regularly for airway clearance and pneumonia prevention in our center. MI-E is used very frequently in our center and is a very important tool for patients with SCI and neuromuscular disease. Typically, MI-E is applied 1–2 times a day for 3–5 sets of 5–6 cycles, depending on the amount of sputum and frequency of suctioning. Assessment of the effectiveness of sputum clearance is conducted to adjust the use of MI-E or other means. However, MI-E is not widely used in general hospitals, where the patients with VPF are first admitted. Home use of MI-E is seldom available in China. Therefore, although patients with VPF could be decannulated and transitioned to non-invasive ventilation support and MI-E, this is not a general practice in China.

The low decannulation failure and reintubation rate did not purely rely on the CF_SV_ threshold but rather on the standardized decannulation protocol [10]. Individualized pulmonary rehabilitation treatment before and after decannulation, especially cough augmentation techniques, for patients with a weak cough ability played an important role [26]. Although the influence of cough augmentation techniques on the rate of successful decannulation and decannulation in critically ill patients was not evident [11], our study suggested that cough augmentation techniques could be important for patients with VPF. If the CF_SV_ value did not reach 100 L/min, the final step of the decannulation protocol would not start. This was different from some studies [27], in which the same patient underwent several attempts and subsequently decannulated.

There was no special description of cough flow measurement methods in many studies, and even though some studies described methods, they were inconsistent [17, 28]. It is important to standardize these measurements and start using them routinely in the decannulation processes [29]. In some studies, the measuring instrument was directly connected to the tracheotomy tube [4, 5, 23]. In our study, we deflated the cuff, and the patients wore the speaking valve to allow measurement of the CF_SV_ from the mouth. We consider it a normal cough process facilitated by a speaking valve [30]; the CF_SV_ was higher than that in Linda’s study [27]. In their study, the optimal cutoff value for predicting successful decannulation was only 29 L/min, but the patients were a neurosurgical cohort whose cough strength was influenced by neurological status. The other reason for the difference was that measurements in their study were generated from a noncuffed tube or a tracheotomy tube with a deflated cuff. The mean PEF with the cuff deflated and a one-way valve was 38% greater than when coughing through the tracheostomy tube, and air leakage during measurements was unavoidable [31].

There are two limitations of this study. The study was conducted in a single center. It is necessary to validate the decannulation protocol in multiple centers. In addition, although many VPF patients were included, the majority were SCI patients and the number of patients with neuromuscular disease was small. The sample size needs to be further expanded to prove whether this standard is sufficient for patients with neuromuscular diseases. And the leak around the tube when coughing necessarily underestimates the actual cough flows before decannulation.

## Conclusion

CF_SV_ greater than 100 L/min is a reliable criterion to successfully decannulate patients with prolonged placement of a tracheostomy tube. The measurement methods should be standardized and used routinely in decannulation processes. For patients with ventilatory pump failure decannulated despite having little to no ability to breathe or vital capacity, it is the MI-E exhalation flows that always exceed 100 L/m [13, 22, 25].

## Data Availability

The datasets used and/or analyzed during the current study are available from the corresponding author on reasonable request.

## Abbreviations

CF_SV_: cough flow measured with tracheostomy tube and speaking valve
VPF: ventilatory pump failure
ABI: acute brain injury
APACHE II: Acute Physiology and Chronic Health Evaluation II
CPF: cough peak flow
GCS: Glasgow Coma Scale
MI-E: mechanical insufflation-exsufflation
MV: mechanical ventilation
PEF: cough peak expiratory flow
SCI: spinal cord injury
